# Our Living World

**Published:** 1885-07

**Authors:** 


Our Living World. An Artistic Edition of the Rev. J. G. Wood's Natural History. Revised by Joseph B. Holder, M.D. New York, published by Selmar Hess.

Students and others interested in Natural History will hail with pleasure the appearance of this interesting work. It is written in good style, the engravings are excellent, and the fact that the services of Dr. Holder have been employed in revising it is a guarantee of its superiority in every respect.

